# ANO1: central role and clinical significance in non-neoplastic and neoplastic diseases

**DOI:** 10.3389/fimmu.2025.1570333

**Published:** 2025-04-28

**Authors:** Yanghao Hu, Yifei Zhang, Jiali He, Huihuang Rao, Duomi Zhang, Zhisen Shen, Chongchang Zhou

**Affiliations:** ^1^ Department of Otorhinolaryngology Head and Neck Surgery, The Affiliated Lihuili Hospital of Ningbo University, Ningbo, Zhejiang, China; ^2^ Health Science Center, Ningbo University, Ningbo, Zhejiang, China

**Keywords:** ANO1, CaCC, diagnostic and therapeutic target, signaling pathways, biomarker

## Abstract

Anoctamin 1 (ANO1), also known as TMEM16A, is a multifunctional protein that serves as a calcium-activated chloride channel (CaCC). It is ubiquitously expressed across various tissues, including epithelial cells, smooth muscle cells, and neurons, where it is integral to physiological processes such as epithelial secretion, smooth muscle contraction, neural conduction, and cell proliferation and migration. Dysregulation of ANO1 has been linked to the pathogenesis of numerous diseases. Extensive research has established its involvement in non-neoplastic conditions such as asthma, hypertension, and gastrointestinal (GI) dysfunction. Moreover, ANO1 has garnered significant attention for its role in the development and progression of cancers, including head and neck cancer, breast cancer, and lung cancer, where its overexpression correlates with increased tumor growth, metastasis, and poor prognosis. Additionally, ANO1 regulates multiple signaling pathways, including the epidermal growth factor receptor (EGFR) pathway, the mitogen-activated protein kinase (MAPK)/extracellular signal-regulated kinase (ERK) pathway, and phosphatidylinositol 3-kinase (PI3K)/protein kinase B (AKT) pathway, among others. These pathways are pivotal in regulating cell proliferation, migration, and invasion. Given its central role in these processes, ANO1 has emerged as a promising diagnostic biomarker and therapeutic target. Recent advancements in ANO1 research have highlighted its potential in disease diagnosis and treatment. Strategies targeting ANO1, such as small molecule modulators or gene-silencing techniques, have shown preclinical promise in both non-neoplastic and neoplastic diseases. This review explores the latest findings in ANO1 research, focusing on its mechanistic involvement in disease progression, its regulation, and its therapeutic potential. Modulating ANO1 activity may offer novel therapeutic strategies for effectively treating ANO1-associated diseases.

## 1 Introduction

Anoctamin 1 (ANO1), also known as TMEM16A, is a calcium-activated chloride channel (CaCC) that belongs to a family of 10 members (A to K, excluding I), each spanning 800 to 1,000 amino acid residues (1). Of this family, ANO1 has been the most extensively studied. The ANO1 gene, located on the human chromosome 11q13 between the cyclin D1 gene (CCND1) - the cortactin gene (EMS1), contains 26 exons (2, 3). The amino acid sequence of ANO1 is composed of 986 residues, with both an intracellular amino terminus and carboxy terminus. The molecular weight of ANO1 is approximately 114 kDa (4).

**Figure 1:**
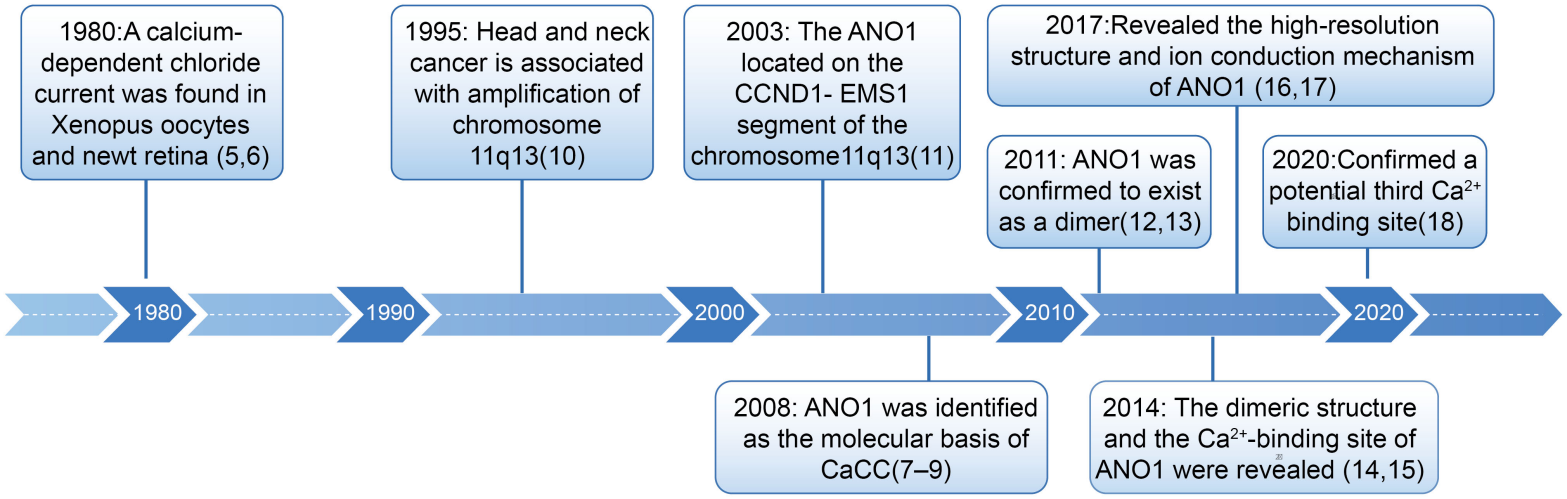
The discover history of ANO1.

**Figure 2:**
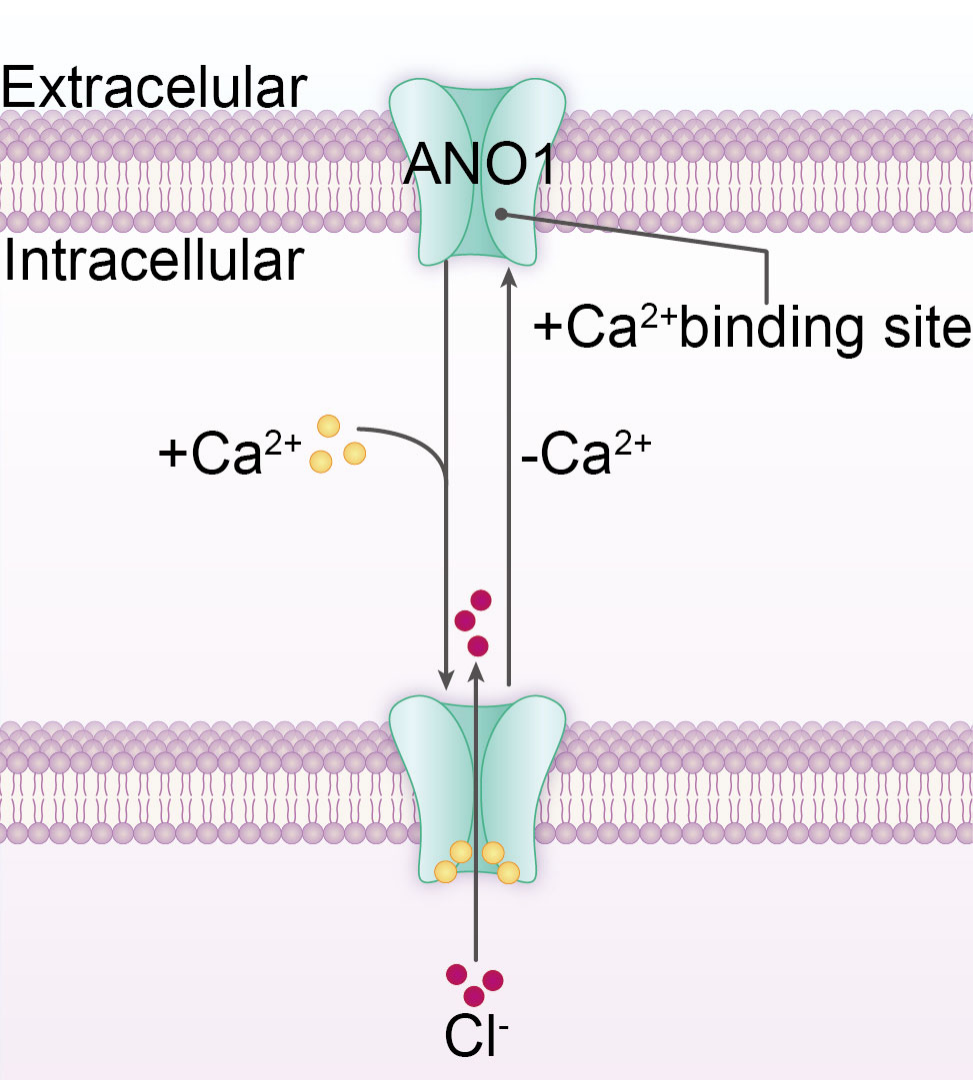
The schematic diagram illustrates the ion conduction mechanism of ANO1. Upon Ca^2+^ binding to the site within the subunit cavity, the cavity undergoes an allosteric change, facilitating ion transport.

In the early 1980s, a calcium-dependent chloride current was identified in Xenopus laevis oocytes and salamander retinas (5, 6). However, the gene encoding this channel remained unknown, hindering subsequent research. It was not until 2008 that three independent research groups simultaneously identified and confirmed that ANO1 constitutes a key component of the CaCC (7–9) (**Figure 1**). This discovery marked a significant milestone in ANO1 research, catalyzing further exploration into its function and underlying mechanisms. ANO1 functions as a homodimer, with each subunit consisting of ten membrane-spanning helices and an extracellular component (16). The subunit also contains a membrane-spanning hydrophilic cleft, referred to as the *subunit cavity*, which spans the entire width of the bilayer and is exposed to the membrane (14). This cavity is sealed from the membrane, effectively blocking lipid entry while enabling selective ion permeation through a gating mechanism involving electrostatic (acidic residues) and steric (hydrophobic gate) elements. This structural feature represents a key distinction between the Anoctamin family ion channels and lipid scramblases (19, 20). The Ca^2+^ binding site is situated in this cavity, one-third of the way through the membrane from the intracellular surface. Ca^2+^ binding induces an allosteric conformational change, facilitating ion conduction (21) (**Figure 2**). Additionally, within the membrane, a spacious cavity (termed dimer cavity) is formed between the transmembrane helices of the two ANO1 subunits, beneath the dimer interface. Directly exposed to the lipid bilayer, this cavity is probably filled by lipids and may contribute to stabilizing the dimer interface (17).

ANO1 is ubiquitously expressed in various native tissues, with particularly high expression levels in pancreatic acinar cells, the retina, renal tubules, and dorsal root ganglia (22). As a CaCC, ANO1 plays a pivotal role in regulating membrane potential and maintaining ion homeostasis, contributing to physiological processes such as epithelial fluid secretion, smooth muscle contraction in the airways and intestines, and mucus secretion in the respiratory tract (23). ANO1 dysfunction has been strongly implicated in several pathological conditions, including asthma (24) and hypertension (25), and has been shown to promote tumor cell proliferation and metastasis in various malignancies, including head and neck squamous cell carcinoma (26), breast cancer (27), and gastric cancer (28). Additionally, ANO1 modulates neuronal sensory conduction and pain regulation (29), and is involved in the pathophysiological mechanisms underlying chronic pain. Extensive research has also established ANO1 as an independent risk factor, highlighting its potential as a diagnostic biomarker for various malignancies and related conditions, offering significant promise for clinical applications (30, 31).

**Figure 3:**
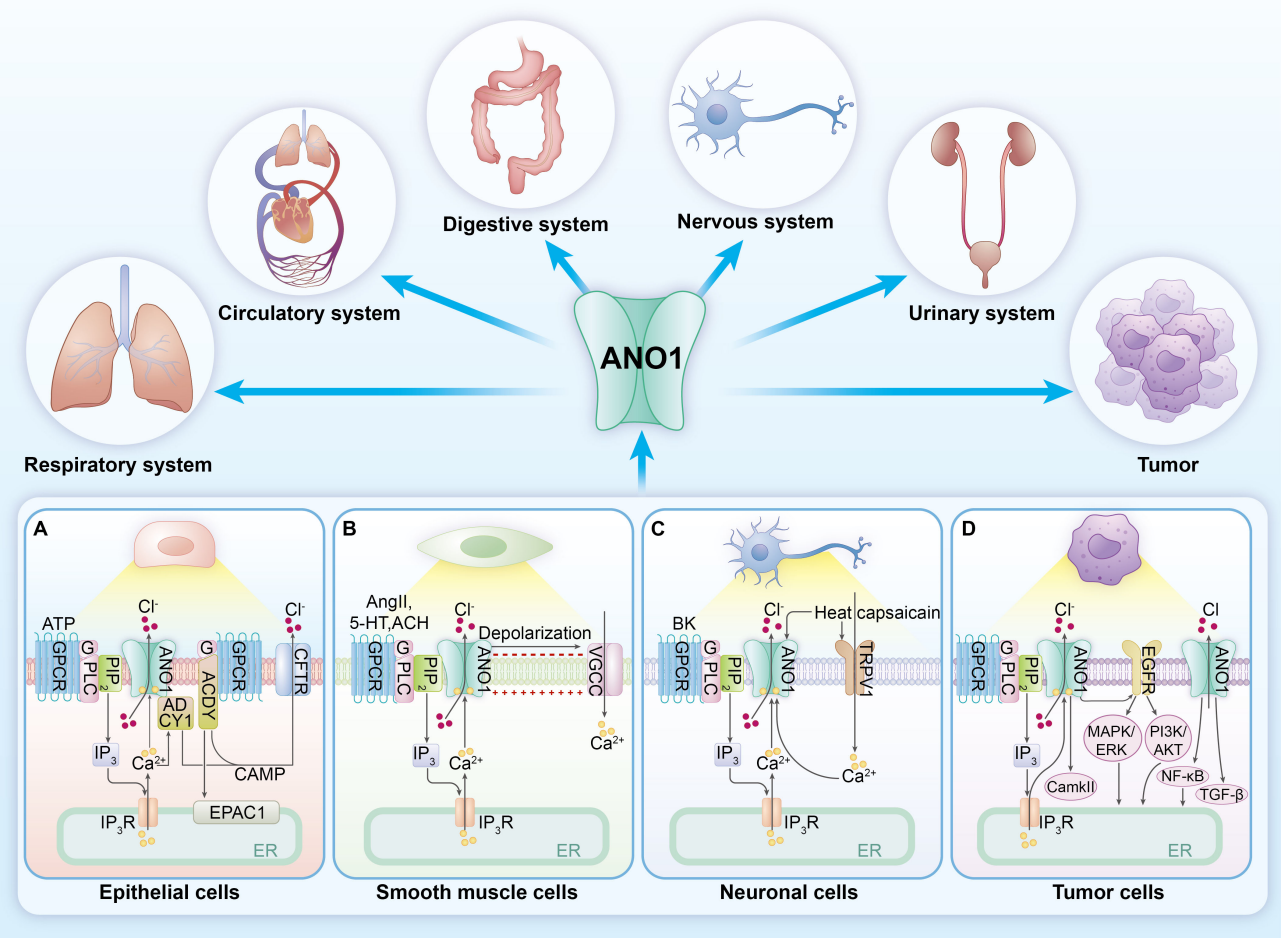
ANO1 in different tissues and its role in diseases. ANO1 is widely expressed across various tissues, and its dysfunction and dysregulation can impact multiple system functions, including the respiratory, circulatory, digestive, and nervous systems. Furthermore, it is closely associated with the onset and progression of tumors. **(A)** In secretory epithelia cells (e.g. airway epithelium cells, Intestinal epithelium), ANO1 and CFTR are localized to the apical membrane. Activation of ANO1 facilitates the transepithelial secretion of Cl^−^ and HCO_3_^−^ into the lumen. Adenosine Triphosphate (ATP) and other ligands activates G protein-coupled receptors (GPCRs), leading to the activation of phospholipase C (PLC), which breaks down membrane phospholipid phosphatidylinositol-4,5-bisphosphate (PIP_2_) into Inositol 1,4,5-trisphosphate (IP3). IP3 induces Ca^2+^ release from the endoplasmic reticulum, elevating intracellular Ca^2+^ levels and subsequently activating ANO1. In airway epithelial cells, activation of GPCRs stimulates adenylate cyclase (ADCY), which generates cAMP and subsequently activating the cystic fibrosis transmembrane conductance regulator (CFTR). ANO1 and CFTR work synergistically in Cl^-^ secretion, with their interaction involving functional crosstalk mediated by Ca^2+^-sensitive adenylate cyclase 1 (ADCY1). **(B)** In smooth muscle cells, angiotensin II (AngII), serotonin (5-HT), and other ligands activate GPCRs, stimulating Ca^2+^ release from the endoplasmic reticulum. This leads to Cl^−^ efflux through ANO1, resulting in membrane depolarization and facilitating Ca^2+^ influx through voltage-gated Ca^2+^ channels (VGCCs), ultimately inducing smooth muscle cell depolarization and contraction. Additionally, AngII can enhance ANO1 expression on the membrane through the PI3K/Akt signaling pathway. **(C)** In neuronal cells, inflammatory mediators increase intracellular Ca^2+^, activating ANO1 and inducing Cl^-^ efflux and neuronal depolarization, which contributes to neuropathic pain. The functional interaction between Transient receptor potential vanilloid 1 (TRPV1) and ANO1 also plays a role in nociception, as both TRPV1 and ANO1 can be activated by heat and capsaicin. **(D)** In tumor cells, ANO1 participates in various signaling pathways, including the EGFR, CaMKII, TGF-β, and NF-κB pathways, playing a critical role in tumor cell proliferation, invasion, and metastasis.

In conclusion, ANO1 is critically involved in the pathogenesis of both tumor and non-tumor diseases, particularly in facilitating tumor invasion and metastasis. This review aims to comprehensively explore the functional roles of ANO1 across both non-neoplastic and neoplastic (**Figure 3**), elucidate the signaling pathways it engages, and assess its potential as a therapeutic target.

## 2 ANO1 and non-neoplastic diseases

### 2.1 Asthma

Asthma is a common inflammatory airway disorder characterized by inflammation, subepithelial fibrosis, hyperplasia of mucus-producing cells, mucus accumulation within airway lumens, hyperplasia of airway smooth muscle (ASM), and ASM hyperresponsiveness (24). The pathogenesis of asthma is complex, and effective treatment options remain limited. Excessive secretion from the airway epithelium and ASM contraction are central drivers of the disease (32). While significant evidence implicates CaCC in these processes, research has been hindered by limited understanding of the underlying genes. The identification of ANO1 as the key gene responsible for CaCC function has recently advanced this field, but its biomolecular and cellular mechanisms in asthma remain incompletely defined.

Mucus is composed of 97% water and 3% solids, with mucin (MUC) proteins being the primary solid component (33). Airway mucus secretion is regulated by various signaling pathways, such as JAG1/Notch/NICD (34), IL-13/STAT6 (35), and IL-8 (36). Despite the diversity of these pathways, evidence indicates that ANO1 plays a role in mucus production in each case (37). Mucus secretion depends on the activation of anion channels to transport Cl^-^ and HCO_3_^-^, which are critical for maintaining the salinity, hydration, and pH balance of the mucus gel layer (32). In asthma, goblet cells primarily secrete the solid components of mucus (38). ANO1 is highly expressed in goblet cells and mediates Ca^2+^-dependent Cl^-^ currents. Moreover, some studies demonstrated that ANO1 exhibits high permeability to HCO_3_^-^ at elevated intracellular Ca^2+^ levels, which may promote mucus release by providing a route for HCO_3_^-^ secretion, thus aiding in mucus dissolution (39). Consequently, ANO1 likely plays a key role in regulating mucus hydration and pH levels. The inhibition of ANO1 expression, either through ANO1 knockout or the use of small molecule inhibitors such as niclosamide, significantly reduces epithelial mucus secretion, suggesting that ANO1 inhibition could be a beneficial strategy for asthma treatment (40).

ASM, a key cellular component in regulating bronchomotor tone, is implicated in airway hyperresponsiveness (41). High expression levels of ANO1 in ASM have been linked to airway hyperresponsiveness (42). Increased ANO1 expression has been observed in both epithelial cells and ASM in allergen-sensitized mouse models (24, 43), suggesting that ANO1 hyperactivity plays a pivotal role in asthma pathogenesis. In addition to the previously discussed pathological changes, chronic airway inflammation plays a significant role in the manifestation of asthma symptoms. Inflammatory mediators such as histamine, prostanoids, cysteinyl leukotrienes, and thromboxane A2 not only directly induce ASM contraction (44) but also bind to their respective G protein-coupled receptors (GPCRs). This binding activates the phospholipase C (PLC)/inositol 1,4,5-triphosphate (IP3) signaling pathway, which triggers the release of Ca^2+^ from the endoplasmic reticulum. The released Ca²^+^ activates ANO1, leading to Cl^-^ efflux and membrane depolarization, which activates voltage-gated Ca^2+^ channels (VGCCs), facilitating additional Ca^2+^ influx. This influx subsequently activates myosin light chain kinase, leading to ASM contraction (45, 46). Recent studies have highlighted the pivotal role of ANO1 in ASM contraction and airway hyperresponsiveness in asthma, acting through the GPCRS/ANO1/VGCCS axis and enhanced cholinergic responses. Furthermore, ANO1 knockdown effectively inhibits CaCC activation and ASM contraction induced by inflammatory mediators (47).

Current asthma management primarily involves the use of inhaled corticosteroids to reduce inflammation, combined with long-acting β_2_-adrenergic agonists to promote bronchodilation. However, 30-45% of patients experience minimal improvement in lung function, and exacerbations continue to pose a significant challenge (41, 48). Consequently, the targeted inhibition of ANO1, by reducing epithelial cell secretion and suppressing ASM contraction, has emerged as a novel potential therapeutic strategy, offering an alternative to conventional treatments. Niclosamide, an FDA-approved anti-parasitic drug, has been identified as a potent ANO1 inhibitor, in addition to its traditional parasiticidal effects. It effectively suppresses ASM depolarization and contraction (49). Cabrita et al. demonstrated that niclosamide mitigates bronchoconstriction, reduces mucus secretion, and exhibits anti-inflammatory effects in asthma models (50), suggesting its promising potential for asthma treatment. Moreover, Huang et al. identified benzbromarone, a clinically used gout medication, as another potential ANO1 inhibitor through high-throughput screening. In an ovalbumin-sensitized mouse model, benzbromarone effectively inhibited ASM contraction and hyperresponsiveness (24). Subsequent studies showed that benzbromarone exhibited similar efficacy to niclosamide in reducing mucus secretion (51). Notably, compared to the commonly used β-agonist isoproterenol, ANO1 inhibitors provided full bronchodilation of airways under conditions of maximal airway contraction or cytokine cocktail pre-treatment, while isoproterenol only partially alleviated airway constriction (49). This highlights the therapeutic advantages of ANO1 inhibitors over traditional β-agonists. However, many ANO1 inhibitors, including T16Ainh-A01, N-(4-methoxy-2-naphthyl)-5-nitroanthranilic acid (MONNA), and idebenone (52–54), show limited efficacy and poor selectivity for asthma treatment. In contrast, Ani9, developed by Yohan Seo and colleagues, demonstrates significantly higher selectivity for ANO1, without affecting related family members, particularly Anoctamin 2, which shares a high amino acid homology to ANO1. This selectivity makes Ani9 a promising candidate for both ANO1 research and therapeutic use (55).

### 2.2 Cystic fibrosis

Cystic fibrosis (CF) is an autosomal recessive disorder primarily caused by mutations in the cystic fibrosis transmembrane conductance regulator (CFTR) gene (56, 57). CFTR is a cyclic adenosine monophosphate (cAMP) dependent anion channel located on the apical membrane of epithelial cells in the airway epithelium, where it plays a pivotal role in Cl^−^ secretion (58). Animal models lacking CFTR exhibit clinical features that closely resemble those of patients with CF, including meconium ileus and exocrine pancreatic destruction (59, 60). Additionally, CFTR-deficient individuals display airway mucus accumulation and obstruction (61), congenital airway abnormalities (62), a reduction in airway surface liquid pH, diminished lysozyme activity, and impaired mucociliary transport (63). These dysfunctions contribute to chronic airway inflammation and accelerate disease progression.

In recent years, there has been significant progress in developing small molecule drugs targeting CF (64, 65), which aim to modulate CFTR function, correct protein misfolding, and increase the open probability of the CFTR ion channel (66). These therapies partially restore defective anion transport, reduce mucus viscoelasticity and concentration, and improve respiratory function (67). These advances have significantly improved the prognosis of patients with CF, leading to long-term survival benefits. However, these therapies are ineffective against mutations that severely disrupt CFTR structure, and further long-term studies are required to assess their efficacy fully in restoring mucus and ciliary clearance functions (66, 68). As a result, there is an urgent need to identify novel therapeutic targets to bypass CFTR defects and address the diverse treatment needs of patients with CF.

ANO1 functions as a CaCC independent of CFTR. Research indicates that specific ANO1 gene knockout results in CF-like pulmonary phenotypes, including tracheal cartilage collapse, mucus obstruction, and defective mucociliary clearance (69), suggesting that ANO1 may play a significant role in the pathogenesis of CF. Current studies suggest that CFTR and ANO1 work together to mediate the secretion of Cl^-^ and HCO_3_^-^ in the airway epithelium (40). Benedetto et al. demonstrated that ANO1 knockout inhibits both Ca^2+^-activated Cl^−^ currents (CaCCs) and CFTR-mediated cAMP-dependent Cl^-^ secretion, and reduces CFTR membrane expression (70). This research also shows that ANO1 engages in functional crosstalk with CFTR through the PSD-95/Dlg/AO-1 protein, explaining their functional overlap (70). Furthermore, studies have revealed that the functional interaction between ANO1 and CFTR is regulated by Ca^2+^-sensitive adenylate cyclase 1 and cAMP-activated sensor proteins (71). These findings highlight that ANO1 can provide a CFTR-independent pathway for Cl^−^ secretion, compensating for the ion secretion defects caused by CFTR mutations in epithelial cells. Currently, several ANO1 activators have been developed for potential CF therapy. Danahay et al. introduced an ANO1 activator, EXT001, which enhances mucociliary clearance in *in vivo* sheep models without affecting calcium mobilization (72). Another ANO1 activator, ET000516-A-2, has also shown promise in improving outcomes for patients with CF (57). These findings support the potential therapeutic benefit of ANO1 activators in CF treatment. Therefore, ANO1 activators offer a novel treatment strategy independent of the CFTR gene for patients with mutations that are not eligible for CFTR modulators, demonstrating potential clinical applicability.

Chronic bacterial airway infections and neutrophilic inflammation are hallmark features of advanced CF lung disease (63), both of which contribute to ANO1 upregulation (24, 73). However, ANO1 expression is primarily upregulated in mucus-producing cells, with minimal expression in ciliated cells during inflammation (40). Activation of ANO1 has been shown to induce bronchoconstriction and is associated with mucus-producing cells, potentially exacerbating respiratory complications (74, 75). In contrast, ANO1 inhibitors reduce mucus production, induce bronchodilation, and restore the mucus-fluid balance in CF airways. These findings suggest that ANO1 inhibition, rather than activation, may offer a more effective therapeutic approach for CF lung disease (76). This raises concerns about strategies aimed at stimulating ANO1 upregulation in patients with CF, which may appear paradoxical. However, some studies present alternative views. For instance, Simões et al. demonstrated that ANO1 upregulation depends on cell proliferation, although ANO1 plays a crucial role in airway secretion, its upregulation is not causally linked to MUC production, as their upregulation occurs independently under different regulatory mechanisms (77). Furthermore, Danahay et al. reported that in their sheep model, ANO1 activation did not induce bronchoconstriction or vascular smooth muscle contraction (72). In addition, recent studies suggest that Brevenal, a potential ANO1 activator, can enhance ANO1 sensitivity to Ca^2+^. However, *in vivo* experiments indicated that it induces mucus release and bronchoconstriction, leading to severe respiratory distress, thus questioning its suitability for inflammatory airway diseases (78). Conversely, another study suggested that Brevenal may improve tracheal mucus transport and alleviate bronchoconstriction (79). These studies suggest that ANO1 can function as a CFTR-independent Cl^−^ secretion pathway to compensate for epithelial secretion deficiencies caused by CFTR mutations, but its excessive activation may exacerbate mucus secretion and bronchoconstriction, increasing the risk of airway obstruction. Preclinical studies have shown conflicting results regarding the efficacy and safety of ANO1 activators, indicating that their application requires careful evaluation. Future research should further explore the regulatory mechanisms of ANO1 and clarify the suitability of its targeted interventions.

### 2.3 Circulatory system

Hypertension, a prevalent cardiovascular disorder affecting a significant global population, increases the risks of comorbidities such as heart disease, stroke, and renal failure due to the prolonged high force of blood against vessel walls (80). Despite the availability of antihypertensive drugs targeting various molecular pathways, therapeutic resistance remains a major challenge. In some cases, even the combination of three or more antihypertensive classes fails to effectively control blood pressure, highlighting the urgent need for novel pharmacological strategies to overcome the limitations of current therapies (81).

Vascular smooth muscle cells (VSMCs) proliferation and remodeling, alongside endothelial dysfunction, are essential in the onset and progression of hypertension (82). VSMCs play a vital role in vascular function, regulating blood flow and pressure and contributing to vessel wall biosynthesis, proliferation, and contraction (83). ANO1, initially identified as a CaCC in pulmonary artery smooth muscle cells (PASMCs), facilitates Cl^−^ efflux and membrane depolarization, thereby activating VGCCs, leading to PASMCs contraction and ultimately increasing pulmonary vascular resistance (84). Further studies have shown that high ANO1 expression is implicated in pulmonary hypertension (PH) development, as well as VSMCs proliferation and pulmonary vascular remodeling. ANO1 inhibitors suppress VSMCs contraction and reverse PH-induced vascular remodeling (85, 86). Similarly, in spontaneous hypertensive rats (SHR), ANO1 overexpression stimulates VSMCs proliferation and vascular remodeling and enhances Ca^2+^ influx, contributing to increased peripheral resistance and hypertension (25). Pharmacological inhibitors targeting ANO1 can block vasoconstriction, thus reducing blood pressure in SHR models (87). Beyond VSMCs, ANO1 overexpression is associated with endothelial dysfunction. It has been shown to upregulate Nox2-containing NADPH oxidase, a key regulator of endothelial dysfunction, which plays a significant role in the pathogenesis of hypertension (88). Moreover, ANO1 overexpression diminishes nitric oxide production and impairs acetylcholine-mediated vasodilation (89). These findings suggest that ANO1 not only directly modulates VSMCs function but also influences endothelial function, affecting vascular tone and blood pressure regulation. The multifaceted role of ANO1 in both smooth muscle and endothelial cells highlights its potential as a promising therapeutic target in hypertension and vascular diseases.

Humoral factors play a pivotal role in the pathophysiology of hypertension, with the activation of the renin-angiotensin-aldosterone system (RAAS) being central to both the onset and progression of the disease (90). Angiotensin II (Ang II), a key mediator of RAAS, is significantly elevated in hypertensive patients (91). Studies have shown that Ang II enhances ANO1 expression in VSMCs through the phosphatidylinositol 3-kinase (PI3K)/protein kinase B (AKT) signaling pathway. This Ang II-induced ANO1 overexpression increases VSMCs sensitivity to additional agonists, thus exacerbating hypertension (25). Conversely, ANO1 also modulates Ang II signaling, promoting the Ras homolog family member A (RhoA)/Rho-associated protein kinase (ROCK) pathway, which leads to the phosphorylation of myosin light chains and the myosin phosphatase-targeting subunit in VSMCs, facilitating their contraction and remodeling (91). This reciprocal regulation between ANO1 and humoral factors underscores a potential therapeutic avenue for managing hypertension.

Furthermore, ANO1 is expressed in various blood vessel types, including coronary (92), basilar (93), and retinal arteries (94). In coronary arteries, MONNA has been shown to effectively reduce membrane depolarization, enhancing coronary blood flow and improving myocardial perfusion (92). Similarly, Ma et al. suggest that ANO1 inhibition contributes to chrysin-induced coronary relaxation by reducing CaCCs (95). Notably, mice lacking ANO1 exhibit lower systemic blood pressure and a diminished hypertensive response to vasoconstrictor treatment (80). These findings emphasize the critical role of ANO1 in modulating vascular contraction and maintaining blood pressure homeostasis, making it a promising target for hypertension and other vascular diseases. However, Wang et al. report that ANO1 expression is downregulated in basilar artery smooth muscle cells (96). Although no ANO1-targeting drugs have yet been clinically approved, growing evidence highlights its therapeutic potential in vascular diseases. While ANO1 inhibitors hold promise, further research is needed to elucidate their precise mechanisms of action and identify the optimal conditions for their therapeutic application.

### 2.4 Digestive system

Diarrhea is a prevalent gastrointestinal (GI) disorder characterized by an increased frequency of bowel movements and the presence of loose or watery stools (97). It arises from an imbalance between intestinal epithelial secretion and absorption, triggered by various factors such as pathogenic infections, allergens, disruptions in bile acid homeostasis, and adverse drug effects (98). The fundamental mechanism driving diarrhea is an increase in Cl^-^ secretion (98). For example, secretory diarrhea caused by cholera results from the overactivation of CFTR in intestinal epithelial cells. Cholera toxin induces excessive activation of adenylate cyclase, leading to the accumulation of cAMP, which drives CFTR-dependent Cl^-^ and water secretion, culminating in diarrhea (99).

In addition to CFTR, recent research has highlighted the role of ANO1 as a CaCC in the pathogenesis of diarrhea. Excessive activation of ANO1 in the intestinal epithelium is closely linked to the pathogenesis of diarrhea (100). ANO1 upregulation has been observed in animal models of diarrhea. Studies have shown that knockdown of ANO1 reduces Ca^2+^-induced Cl^−^ secretion in intestinal epithelial cells (25, 101). Furthermore, ANO1 upregulation has been reported in rotavirus-induced diarrhea (101, 102), where non-structural protein 4 (NSP4) elevates intracellular Ca^2+^ and activates the PLC/IP3 signaling pathways, enhancing epithelial secretion and promoting diarrhea (103, 104). The use of ANO1 inhibitors has been shown to mitigate watery stools and fluid loss in rotavirus-infected models (105). Additionally, several natural products have been identified as effective ANO1 inhibitors. Tannic acid, found in green tea and red wine, blocks ANO1, reducing intestinal chloride secretion, making it a potential therapeutic agent for secretory diarrhea (106). The FDA-approved drug Crofelemer, derived from *Croton lechleri*, inhibits both CFTR and ANO1 and is clinically used for the treatment of HIV-related diarrhea. Its high stability, prolonged efficacy, and favorable safety profile further highlight its therapeutic potential (107, 108).

Increased GI tract activity is another contributing factor to diarrhea (109). Interstitial cells of Cajal (ICCs) in the GI smooth muscle are mesenchymal cells that play a critical role in regulating muscle contractions (110). They generate rhythmic membrane potential oscillations, modulate the propagation of slow waves, promote muscle contraction, and stabilize the resting membrane potential in smooth muscle cells (110, 111). ANO1 is predominantly expressed in ICCs within the GI tract (112) and plays a pivotal role in mediating slow wave current generation, as Ca^2+^ release and T-type current activation facilitate voltage-dependent Ca^2+^ influx into ICCs. ANO1 amplifies slow wave depolarization, enhancing its propagation across the ICCs network (110). Targeted knockout of ANO1 leads to the loss of slow waves and induces irregular-amplitude oscillations, suggesting that inhibiting ANO1 could reduce GI tract activity (113, 114). Both ANO1 inhibition and knockout impair slow-wave activity. Several ANO1 inhibitors, such as niflumic acid and 5-nitro-2-(3-phenylpropylamino) benzoic acid, have been shown to suppress slow waves (114, 115). These inhibitors may exhibit varying effects due to differences in target tissues, but the underlying mechanisms remain to be fully elucidated (116). Overall, these studies underscore the critical role of ANO1 in the pathological mechanisms of diarrhea, by simultaneously suppressing epithelial cell secretion and GI smooth muscle activity, may serve as a promising therapeutic strategy for secretory diarrhea.

ANO1 is implicated in various digestive system diseases, playing a critical role in bile formation and secretion by facilitating the secretion of HCO_3_^-^ and Cl^-^ in cholangiocytes. Dysfunctional ANO1 can impair bile secretion, promoting gallstone formation, suggesting its potential as a susceptibility gene for gallstones (117, 118). Under hypoxic conditions, downregulation of ANO1 activity has been shown to increase bile toxicity, contributing to cell death (119). Additionally, ANO1 is strongly associated with epithelial proliferation in eosinophilic esophagitis. ANO1 expression in the esophageal basal zone correlates positively with disease severity and basal zone hyperplasia. Disrupting IL-13-induced Cl^-^ transport *via* pharmacological inhibition or gene silencing effectively attenuates esophageal epithelial proliferation (120). In acute pancreatitis, ANO1 may increase intracellular Ca^2+^ levels and stimulate IL-6 secretion. IL-6 upregulates ANO1 expression through the IL-6/STAT3 pathway, and ANO1 overexpression further enhances IL-6 secretion in pancreatic acinar cells *via* the IP3/Ca^2+^/nuclear factor kappa-B (NF-κB) pathway. This cascade may contribute to the pathogenesis of acute pancreatitis, suggesting that inhibiting ANO1 could be a promising therapeutic strategy (121). Furthermore, ANO1 regulates glucose-induced insulin secretion in the pancreas (122, 123). In summary, ANO1 plays a multifaceted role in the digestive system, and its dysregulation significantly contributes to the pathophysiology and progression of various diseases. As a key factor in these conditions, ANO1 offers valuable opportunities for the development of innovative diagnostic and therapeutic strategies.

### 2.5 Nervous system

Pain results from heightened sensory excitability due to injury or disease, manifesting as allodynia to non-noxious stimuli or increased sensitivity to harmful stimuli (124). This phenomenon can be attributed to peripheral or central sensitization, involving enhanced synaptic excitation, reduced synaptic inhibition, increased neuronal responsiveness, or a combination of these factors (125, 126). Pain signaling occurs through sensory neuron clusters within the dorsal root ganglia (DRG) of spinal nerves (127). Electrophysiological studies indicate that ANO1 enhances excitability and depolarization in DRG neurons (128). Additionally, anatomical studies indicate that ANO1 is predominantly expressed in small-diameter DRG neurons, with significant colocalization with Transient receptor potential vanilloid 1(TRPV1), which is involved in nociception (129, 130). Both ANO1 and TRPV1 are heat sensors, activated by noxious heat above 44°C and 43°C, respectively. Studies show that ANO1 knockout in DRG neurons reduces nociceptive behaviors in thermal pain models, highlighting its role in pain sensation (129, 131, 132). Inflammatory mediators, such as bradykinin, also contribute to pain by activating B2 receptors and PLC, which inhibit M-type K^+^ channels while opening CaCCs. This leads to membrane depolarization in sensory afferents, triggering pain signals. Pharmacological inhibition of ANO1 or activation of M-channels can reduce pain behaviors, further validating ANO1 as a promising therapeutic target for pain management (128). Studies have shown that the B2 receptor functions as a GPCR (128), with ANO1 channels being selectively activated by intracellular Ca^2+^ release induced by GPCRs, rather than by VGCCs (29). This selective activation is dependent on specific domain interactions between the ANO1 plasma membrane and the perinuclear endoplasmic reticulum, allowing targeted responses to distinct calcium signals from inflammatory mediators while avoiding interference from overall intracellular calcium fluctuations (29). Furthermore, ANO1 expression is upregulated in mouse spinal cord injury models, and ANO1 inhibitors effectively reduce both mRNA and protein levels of ANO1 (133). Additionally, ANO1 inhibitors significantly decrease capsaicin-induced currents and action potentials, while also mitigating nociceptive behaviors in mice (134). Together, these findings suggest that ANO1 plays a significant role in the pathophysiology of pain, and targeting ANO1 holds considerable potential as a therapeutic strategy for pain management.

### 2.6 Urinary system

Urethral smooth muscle generates tension through contraction, playing a critical role in preventing urinary incontinence. This urethral tone is maintained by the coordinated contraction of urethral smooth muscle cells (USMCs), which prevents urine leakage during bladder filling. However, studies show species differences variability in the expression of the ANO1 channel in USMCs and urethral interstitial cells (UICCs). For instance, rabbit studies indicate ANO1 expression in UICCs, influencing USMCs excitability (135), whereas this has not been observed in mice (42). Other studies report ANO1 expression in USMCs of mice, rats, and sheep, with no expression in UICCs (136). These discrepancies may be attributed to differences in antibody selection or sample preparation, underscoring the need for further research to elucidate the causes of these variations (137). Despite these inconsistencies, ANO1 undeniably plays a pivotal role in regulating spontaneous tonic tension (STT) in the urethra. Higher ANO1 expression in females may be involved in the increased prevalence of stress urinary incontinence in this population (138). Furthermore, hypertriglyceridemia reduces STT and urethral contractility by downregulating ANO1 expression, thereby impairing urinary function (139). These findings suggest that ANO1 holds promise as a therapeutic target, offering potential strategies for treating urinary incontinence and related disorders.

## 3 ANO1 and neoplastic diseases

### 3.1 Head and neck squamous cell carcinoma

Head and neck squamous cell carcinoma (HNSCC) is the sixth most prevalent cancer globally, with over 600,000 new cases annually, of which nearly 45% present with regional lymph node metastasis at the time of diagnosis (140). Despite significant advancements in HNSCC diagnosis and treatment, the late-stage diagnosis and associated risk behaviors result in a five-year survival rate that remains distressingly below 50% (141, 142). Thus, identifying effective drug targets and predictive biomarkers for HNSCC is essential for improving patient prognosis. Studies have shown that ANO1 overexpression in HNSCC activates the mitogen-activated protein kinase (MAPK)/extracellular signal-regulated kinase (ERK) signaling pathway, promoting tumor proliferation and correlating with poor prognosis (143). Knockdown of ANO1 in HNSCC reduces MCL1 expression, redistributes p27^Kip1^ to the nucleus and perinuclear region, induces cell cycle arrest, inhibits tumor proliferation, and promotes apoptosis (26). However, Shiwarski et al. reported that downregulation of ANO1 enhances HNSCC cell motility and increases metastasis, independent of 11q13 amplification (144). These conflicting findings highlight the complex role of ANO1 in HNSCC progression. Further studies reveal that ANO1 expression is closely linked to tumor subtypes, with high expression correlating with shorter survival and distant metastasis in HNSCC (145, 146), whereas no prognostic significance is observed in HPV-positive HNSCC (146). This suggests that ANO1 may not be a viable therapeutic target for this subgroup. These findings imply that the function of ANO1 is influenced by the tumor microenvironment and specific tumor cell types, warranting further clinical and molecular research to clarify its role in cancer progression.

Platinum-based chemotherapy is a cornerstone of first-line treatment for various cancers (147). The integration of platinum-based drugs with other chemotherapeutic agents and immunotherapy has significantly improved survival rates in patients with HNSCC (148). Recent studies suggest that ANO1 contributes to tumor cell drug resistance. Vyas et al. demonstrated that ANO1 overexpression increases oxidative stress, which upregulates ATPase copper-transporting beta, leading to the sequestration of platinum-based drugs within lysosomes, thereby conferring drug resistance (149). Notably, copper chelators can reverse ANO1-mediated resistance, providing valuable therapeutic insights for HNSCC treatment (149). Furthermore, ANO1 can aid in risk stratification and optimization of chemotherapy regimens in HNSCC. Studies show that patients with cisplatin-resistant oral squamous cell carcinoma in the high-risk group exhibit greater sensitivity to docetaxel and shikonin compared to the low-risk group, while both groups demonstrate similar sensitivity to roscovitine (150). Thus, ANO1 plays a critical role in guiding treatment decisions, offering valuable insights into drug sensitivity that can help optimize therapeutic strategies.

### 3.2 Breast cancer

Breast cancer accounts for 11.6% of all cancers, making it one of the most prevalent malignancies worldwide and a leading cause of cancer-related mortality among women (6.9%) (151). Studies have shown that 11q13 amplification is strongly associated with poor prognosis in patients with breast cancer (152). Further investigations revealed that ANO1 amplification and overexpression are significantly correlated with tumor grade and poor prognosis, promoting tumor proliferation through the epidermal growth factor receptor (EGFR) and calmodulin-dependent protein kinase II (CAMKII) signaling pathways (27). Additionally, ANO1 expression is closely associated with immunohistochemical markers such as β-catenin, cyclin D1, and E-cadherin, and has been identified as an independent prognostic marker in breast cancer (153). Inhibition of ANO1 induces G0/G1 cell cycle arrest and significantly reduces the invasiveness of breast cancer cells (153). Collectively, these findings underscore the critical role of ANO1 in breast cancer progression and its potential as a therapeutic target. Key biomarkers in breast cancer, including Human Epidermal Growth Factor Receptor 2 (HER2), progesterone receptor (PR), and estrogen receptor (ER), are central to treatment strategies (154). Endocrine therapy and anti-HER2 targeted treatments, based on these markers, have significantly improved patient prognosis and quality of life (155). Wu et al. demonstrated a notable association between ANO1 expression and HER2, PR, and ER positivity in breast cancer (156). In contrast, Zhang et al. indicated that ANO1 is not associated with PR or ER in breast cancer (157). These conflicting findings highlight the complex role of ANO1 in breast cancer, suggesting that its function may vary across different cellular subtypes and cell lines. Further research is required to clarify its relationship with hormone receptors and its prognostic significance in specific cellular subtypes, thus providing new insights for precision medicine.

### 3.3 Colorectal cancer

Colorectal cancer (CRC) is one of the most prevalent malignancies, representing 9.6% of all cancers and ranking as the third leading cause of cancer-related mortality globally (9.3%) (151). ANO1 upregulation has been linked to tumor progression and higher TNM staging in CRC (158). Sui et al. demonstrated that ANO1 knockout inhibits the growth, migration, and invasion of CRC cells by suppressing the MAPK/ERK signaling pathway (159). Recent studies have also shown that endogenous knockout of ANO1 inactivates downstream Wnt/β-catenin signaling, thereby inhibiting cell proliferation and inducing apoptosis in CRC, consistent with prior findings (160). Li et al. suggested that ANO1 mRNA expression is strongly correlated with lymph node metastasis in CRC, serving as a critical independent predictor for lymph node involvement (161). These findings highlight ANO1 as a key player in CRC progression and metastasis, positioning it as both a potential therapeutic target and prognostic biomarker, with promising implications for future treatment strategies.

### 3.4 Prostate cancer

Prostate cancer accounts for 7.3% of all cancers globally, ranking as the fourth most common malignancy worldwide (151). Studies have shown that overexpression of ANO1 is closely associated with TNM staging and Gleason scores in prostate cancer, playing a critical role in the proliferation, progression, and metastasis of the disease (162). Silencing or inhibiting endogenous ANO1 has been demonstrated to suppress prostate cancer growth, induce apoptosis, and enhance TNF-α expression (163). Additionally, some studies have shown that natural products such as resveratrol and luteolin can act as ANO1 inhibitors while also exhibiting anti-tumor activities, effectively suppressing the proliferation and migration of prostate cancer cells (164, 165). Collectively, these findings suggest that ANO1 represents a promising anticancer target in prostate cancer therapy.

### 3.5 Ovarian cancer

Globally, ovarian cancer is the most lethal gynecological cancer, with the eighth highest incidence and fifth highest mortality rate among female cancers (166). Among gynecologic malignancies, epithelial ovarian cancer ranks as the second leading cause of death. Its treatment remains challenging due to nearly 75% of cases being diagnosed at advanced stages (167). Studies have shown that ANO1 is significantly overexpressed in epithelial ovarian cancer cells, with its upregulation strongly associated with higher FIGO (International Federation of Gynecology and Obstetrics) stages and lower differentiation grades (168). Furthermore, downregulation of ANO1 reduces PI3K/Akt phosphorylation, inhibiting ovarian cancer cell growth by disrupting the PI3K/Akt signaling pathway (168). Therefore, ANO1 plays a critical role in ovarian cancer progression, correlating with poor prognosis and advanced stages. Its regulation of the PI3K/Akt pathway highlights its potential as a valuable therapeutic target.

### 3.6 Others

ANO1 is also closely associated with various other tumors, including gastrointestinal stromal tumors (GIST), esophageal squamous cell carcinoma (ESCC), and pancreatic ductal adenocarcinoma (PDAC). ANO1, also referred to as gastrointestinal tumor protein 1, is a biomarker of GIST and is commonly used in immunohistochemical diagnostics (169, 170). Studies have shown that ANO1 is significantly amplified in peripheral blood mononuclear cells, with its average expression level and range in the blood increasing in parallel with ANO1 expression in tissues. Furthermore, the level of ANO1 overexpression is associated with tumor size (171). Furthermore, ANO1 expression levels decrease in most patients with GIST after surgery and increase during recurrence (172). Therefore, ANO1 can serve as a biomarker for tumor recurrence monitoring and the assessment of poor prognosis in GIST. Moreover, ANO1 not only promotes ESCC cell proliferation, migration, and invasion through the activation of the transforming growth factor β signaling pathway (173–175), but it also serves as a biomarker of poor prognosis in PDAC. Its overexpression is closely associated with enhanced cell migration, reduced long-term patient survival, and upregulation of the EGFR signaling pathway in PDAC patients (176, 177). Recent research suggests that ANO1 contributes to the development of an immunosuppressive tumor microenvironment (TME), driving cancer cell resistance to anti-PD-1 immunotherapy (178). In pancreatic cancer, ANO1 influences the TME through cytokine and interleukin signaling, promoting fibroblast accumulation and reducing CD8+ T cell infiltration *via* paracrine signaling, thereby creating a TME that facilitates tumor growth and immune evasion (31). Clinically, targeting ANO1 in combination with immune checkpoint inhibitors could enhance therapeutic efficacy (31). As both a biomarker and therapeutic target, ANO1 demonstrates significant clinical value across multiple cancers. It not only aids in optimizing individualized treatment regimens but also enhances therapeutic efficacy through combined inhibition strategies, showing broad potential for clinical application.

## 4 Signaling pathways activated by ANO1 in cancer

**Figure 4:**
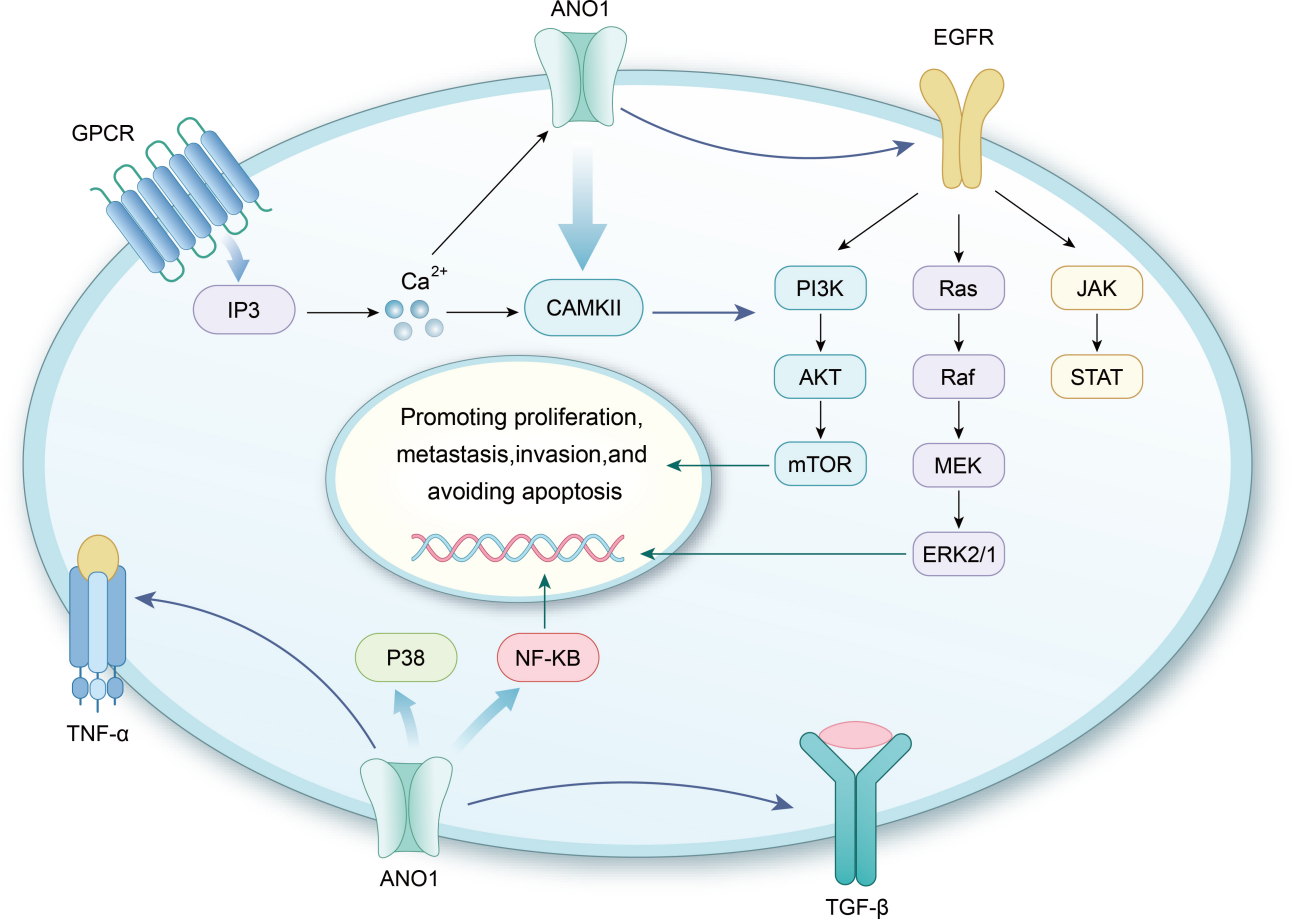
ANO1 participates in various signaling pathways. ANO1 directly or indirectly interacts with EGFR, promoting EGFR phosphorylation and activating the MAPK/ERK, PI3K/Akt, and JAK/STAT signaling pathways. Additionally, GPCRs interacts with IP3, leading to the release of Ca^2+^ from the endoplasmic reticulum. This increases intracellular calcium levels, activating CaMKII, which subsequently activates the aforementioned signaling pathways. In head and neck cancer, ANO1 activation is associated with the MAPK/ERK signaling pathway. In gliomas, ANO1 activates the NFκB signaling pathway. Furthermore, ANO1 interacts with the TNF-α and TGF-β signaling pathways, which are closely linked to tumor proliferation, invasion, metastasis, and poor prognosis.

ANO1 contributes to tumor proliferation and progression through various signaling pathways, as illustrated in **Figure 4**.

### 4.1 ANO1 and EGFR signaling pathway

EGFR, a tyrosine kinase receptor commonly overexpressed in tumors like HNSCC and breast cancer, plays a pivotal role in tumor progression (179). In HNSCC, ANO1 promotes cell proliferation by enhancing EGFR phosphorylation and expression, as well as forming a complex with EGFR (180). In breast cancer, ANO1 inhibition suppresses EGFR phosphorylation, reduces the activity of downstream signaling molecules such as AKT and ERK, and diminishes the autocrine secretion of EGFR ligands, including EGF and transforming growth factor α. These findings highlight ANO1’s critical role in enhancing EGFR pathway activation (27). Thus, ANO1 regulates EGFR signaling by promoting EGFR expression, phosphorylation, and autocrine secretion.

### 4.2 ANO1 and CAMKII signaling pathway

Ca^2+^/calmodulin(CaM)-dependent protein kinases (CaMKs), versatile serine/threonine kinases, are regulated by Ca^2+^ signaling (181). Elevated levels of various CaMKs isoforms, particularly CAMKII, have been observed in several cancers (27, 182). Britschgi et al. showed that ANO1 overexpression promotes Ca^2+^/CaMKII phosphorylation, suggesting that increased ANO1 levels activate calcium-dependent CaMKII signaling pathways (27). ANO1 channels are activated by calcium release *via* GPCRs and do not respond to calcium influx from VGCCs. This selective activation depends on the interaction of ANO1 with inositol 1,4,5-trisphosphate receptor type 1 (IP3R1) and its anchoring in membrane microdomains, allowing ANO1 to respond specifically to localized calcium signals rather than global intracellular calcium fluctuations (29). Moreover, studies suggest that the ANO1-IP3R1 interaction enhances ATP-induced Ca^2+^ release from the endoplasmic reticulum in HeLa cells, with ANO1 inhibitors significantly reducing ATP-induced Ca^2+^ influx (183). These findings suggest that Ca^2+^ release activates the CaMKII pathway, thereby promoting cancer cell proliferation.

### 4.3 ANO1 and MAPK/ERK signaling pathway

The MAPK/ERK signaling pathway plays a pivotal role in regulating normal cellular proliferation, survival, and differentiation. Dysregulation of this pathway is closely associated with cancer and various other diseases (184). In HNSCC, ANO1-induced cancer cell proliferation and tumor growth are coupled with activation of extracellular-regulated kinases 1/2 (ERK1/2), and inhibition of ERK1/2 effectively reduces ANO1-driven cell proliferation (143). In CRC, ANO1 downregulation results in altered expression of phosphorylated mitogen-activated protein (MEK) and ERK1/2 (159). Upregulation of ANO1 has been shown to stimulate ERK1/2 signaling in breast cancer cells *via* CaMKII- and EGFR-mediated pathways (27). In liver cancer, ANO1 knockdown leads to reduced phosphorylation of p38 and ERK1/2 (185). Recent studies reveal that ANO1 upregulation is associated with activation of the MEK/ERK and AKT pathways in BRAF-mutant melanoma cells, correlating with poor patient prognosis (186). Additionally, arctigenin, a novel ANO1 inhibitor, has been found to inhibit the MAPK pathway in lung adenocarcinoma treatment (187). These findings suggest that ANO1 may modulate cancer cell behavior through the MAPK/ERK signaling pathway.

### 4.4 ANO1 and PI3K/Akt signaling pathway

The PI3K/Akt pathway is a critical regulator of cellular proliferation and survival, with gene mutations in various human cancers reported to enhance PI3K kinase activity. Dysregulation of PI3K frequently results in abnormal Akt activation (188). Study has shown that suppression of ANO1 expression significantly inhibits the phosphorylation of CAMKII, EGFR, Akt, and ERK1/2 in breast cancer, HNSCC, and ESCC cells (27). In ovarian cancer, ANO1 knockdown reduces PI3K/Akt phosphorylation, and the use of specific inhibitors can suppress ANO1-mediated ovarian cancer cell growth by targeting PI3K/Akt signaling (168). Recent evidence indicates that in gastrointestinal tumors, ANO1 promotes tumor progression by inhibiting ferroptosis in a PI3K/Akt-dependent manner (178). These findings suggest that ANO1 contributes to cancer progression through modulation of the PI3K/Akt signaling pathway.

### 4.5 ANO1 and NF-κB signaling pathway

NF-κB is a family of transcription factors involved in regulating inflammation, immune responses, development, and cell survival. Strong evidence links NF-κB to cancer and inflammation, with the pathway playing a pivotal role in immune homeostasis, chronic inflammation, tumorigenesis, and development (189). NF-κB regulates numerous genes associated with immune-inflammatory responses, cell cycle progression, apoptosis inhibition, and cell adhesion, promoting oncogenic processes and cancer progression (190). In glioma cells, overexpression of ANO1 significantly increases the phosphorylation of the NF-κB inhibitor, leading to the nuclear accumulation of the NF-κB subunit p65. Furthermore, luciferase reporter assays reveal that ANO1 overexpression substantially upregulates NF-κB -mediated gene transcription activity. These findings suggest that heightened NF-κB activity is associated with ANO1 overexpression (191). However, the precise mechanisms by which ANO1 activates the NF-κB signaling pathway remain unclear and warrant further exploration.

## 5 Discussion

ANO1 plays a pivotal role in the pathophysiological processes of both non-neoplastic and neoplastic diseases and represents a promising therapeutic target for disease intervention. Furthermore, ANO1 is widely expressed across various malignancies, orchestrating multiple signaling pathways and contributing significantly to tumor initiation and progression. Notably, ANO1 serves as an independent prognostic biomarker closely associated with poor outcomes in multiple cancers, underscoring its clinical utility in tumor assessment and prognostic evaluation.

In this review, we elucidate the critical role of ANO1 in non-neoplastic diseases. Elevated ANO1 expression has been implicated in excessive mucus secretion and abnormal smooth muscle contraction, contributing to the pathophysiology of conditions such as asthma, hypertension, and diarrhea. Given its crucial role in these pathological processes, ANO1 has emerged as a promising therapeutic target. Magnolol alleviates allergic rhinitis by inhibiting ANO1, reducing mucus secretion and inflammatory cytokine release (192). T16Ainh-A01 inhibits ANO1 overexpression-induced proliferation of cardiac fibroblasts and collagen deposition, exhibiting additional anti-cardiac fibrosis effects and potential therapeutic value (193, 194). Similarly, the novel analgesic 4-arylthiophene-3-carboxylic acid inhibits ANO1 with high selectivity, demonstrating therapeutic efficacy comparable to widely used clinical drugs and promising potential in pain management (195). These findings further support the clinical value of ANO1 as a potential therapeutic target. Furthermore, we elucidated the association between ANO1 and a range of neoplastic diseases. Meta-analysis indicates that the overexpression of ANO1 is strongly correlated with poor prognosis and reduced long-term survival in several epithelial-derived malignancies, including breast cancer, HNSCC, ESCC, and CRC (157). In addition, the high expression of ANO1 is closely associated with the advanced clinical stages of prostate cancer and ovarian cancer, as well as poor pathological differentiation grades, suggesting that ANO1 expression levels can serve as an important indicator for predicting poor tumor prognosis (162, 168). Notably, in HNSCC, both high ANO1 protein expression and gene amplification are associated with shorter survival, as ANO1 enhances tumor cell migration, and promotes distant metastasis (196). Additionally, elevated ANO1 expression is accompanied by increased phosphorylation of ERK1/2, activating the MAPK/ERK pathway while upregulating Cyclin D1 expression, thereby facilitating the G1/S phase transition and promoting tumor cell proliferation (143). Recent studies further reveal that high ANO1 expression is associated with poorer overall survival OS in HNSCC. ANO1 overexpression not only serves as an independent prognostic biomarker for poor prognosis in HNSCC, aiding in patient risk stratification and survival prediction, but also plays a critical role in radiotherapy and cisplatin resistance. Targeting ANO1 inhibition can enhance HNSCC sensitivity to chemo-radiotherapy, providing a novel precision treatment strategy for high-risk patients and offering a potential therapeutic target for personalized therapy, such as combined PI3K/EGFR inhibition (197). Moreover, ANO1 is closely associated with the TME. It promotes immune evasion, activates tumor-associated fibroblasts, and shapes an immunosuppressive TME, thereby accelerating tumor growth, invasion, and drug resistance (198). High ANO1 expression also reprograms cholesterol metabolism by inhibiting LXR-mediated cholesterol efflux, leading to intracellular cholesterol accumulation, which enhances cancer cell survival, increases metastatic potential, and is linked to resistance to anti-PD-1 therapy (178, 199). Therefore, ANO1 holds dual value as both a therapeutic target and a biomarker in neoplastic diseases, facilitating patient prognosis assessment, risk stratification, and clinical decision-making, thereby advancing precision medicine and personalized treatment strategies.

However, several challenges need to be addressed. Current small-molecule ANO1 inhibitors may also affect other ion channels and endothelial cell function, leading to nonspecific vasodilation and potential cytotoxicity, which could impair cell proliferation (200). Additionally, these inhibitors can disrupt intracellular calcium homeostasis, resulting in endoplasmic reticulum calcium store depletion or impaired calcium release (201). Such disruptions may lead to smooth muscle dysfunction and secretory abnormalities, potentially causing GI motility disorders, including constipation or intestinal obstruction, as well as impairments in exocrine function, manifesting as reduced secretion of saliva, tears, and gastric fluids. Furthermore, studies have revealed a paradoxical role of ANO1 in PDAC. Unexpectedly, ANO1 exhibits lower expression in highly invasive cancer cells while being more highly expressed in adherent tumor cells (176), which presenting a challenge for ANO1-targeted therapy. Future research should enhance the selectivity and safety of targeted therapy, optimize treatment strategies, and precisely identify suitable patient groups to advance clinical. In recent years, the rapid advancement of artificial intelligence (AI) has significantly facilitated the application of 2D and 3D quantitative structure-activity relationship models in the screening of ANO1 pharmacological modulators, presenting transformative opportunities for the precise design and development of ANO1-targeted therapies (202). As AI technologies and bioinformatics methods continue to evolve, the screening and optimization of ANO1 modulators are becoming more efficient and precise, thereby driving the development of novel therapeutic strategies for related diseases. Overall, ANO1 has emerged as a crucial therapeutic target with great potential in cancer treatment and various disease interventions. However, its targeted therapy still faces challenges such as insufficient selectivity, potential side effects, and patient suitability screening. The integration of AI not only accelerates the development of novel ANO1 modulators but also provides powerful tools for personalized treatment, offering the prospect of more precise and effective clinical applications in the future. Therefore, future research should further integrate AI technologies with experimental validation to advance ANO1-targeted therapies with greater selectivity, safety, and clinical translational value, ultimately achieving breakthrough progress in precision medicine and clinical oncology.

## 6 Conclusions

ANO1, a key CaCC, plays a critical role in both non-neoplastic (**Table 1**) and neoplastic diseases (**Table 2**). In non-cancerous conditions, ANO1 contributes to the pathogenesis of asthma, cystic fibrosis, and hypertension by regulating ion homeostasis and membrane potential, positioning it as a promising therapeutic target. In cancer, ANO1 regulates key signaling pathways, including EGFR, MAPK/ERK, and PI3K/Akt, driving tumor growth and metastasis. Its overexpression is closely linked to poor prognosis and drug resistance, further solidifying its importance as a diagnostic and therapeutic target. However, existing inhibitors are limited by selectivity and efficacy, underscoring the need for more precise drug development. In conclusion, further investigation into the molecular mechanisms of ANO1 and the development of targeted therapies holds significant potential for advancing disease diagnosis and treatment.

**Table 1:** Role of ANO1 in non-neoplastic diseases.

Disease	Regulation of ANO1			ANO1-dependent regulation	Role	Reference
	Stimuli	Mechanism	ANO1 Expression			
Asthma	IL-13	JAK-STAT6/ PI3K-AKT	High	MAPK/ERK pathway / Secretion of Cl^−^ and HCO_3_^−^	Promotes mucus secretion in airway epithelium cell and enhances goblet cell metaplasia	(35, 37, 203, 204)
	IL-4	JAK-STAT6/ PI3K-AKT	High	Secretion of Cl^−^ and HCO_3_^−^		(203, 205)
	Eact	ANO1 activator	High	Depolarization /Ca^2+^	Promotes contraction and hyperresponsiveness of VSMCs	(75)
	5-HT	GPCR/PLC/IP3	High			(47)
Cystic Fibrosis	bacterial components and pyocyanin	Oxidative Stress	High	Secretion of Cl^−^ and HCO_3_^−^/Ca^2+^	Compensates for CFTR deficiency and promotes chloride ion secretion	(73)
Hypertension	Ang II	PI3K/AKt	High	ROCK pathway phosphorylation of MLC and MLCP/ Depolarization /Ca^2+^	Promotes contraction and proliferation of vascular smooth muscle cells	(91)
Diarrhea	Serum and NSP4	GPCR/PLC/IP3	High	Secretion of Cl^−^ and HCO_3_^−^/ Depolarization	Promotes secretion of intestinal epithelial cells and enhances gastrointestinal smooth muscle activity	(101)
Acute Pancreatitis	IL-6	IL-6R/STAT3	High	NFκB pathway	Promotes the expression of inflammatory cytokines and premature activation of trypsinogen	(121)
Eosinophilic Esophagitis	IL-13	JAK-STAT6	High	TP63 Expression and CDK2 Phosphorylation	Promotes the proliferation of esophageal epithelial cells	(120)
Pain	Bradykinin	GPCR/PLC/IP3	High	Depolarization	Triggers action potentials in neurons	(128, 206)
	Heat, Capsaicin,		High	Depolarization		(206)
Urinary Incontinence	Hypertriglyceridemia	Transcriptional regulation	Low	Cl^−^	Spontaneous Tone of urethral smooth muscle is reduced	(139)

**Table 2:** Role of ANO1 in neoplastic diseases.

Disease type	Mechanism	Cell line	Role	Reference
Colorectal cancer	ERK1/2	HCT8, HCT116, SW480, SW620, LS174T	Participate in growth, migration, and invasion of metastatic CRC cells	(159)
Breast cancer	EGFR and STAT3 phosphorylation	MCF-7, T47D	Promote breast cancer cell proliferation and tumor growth	(207)
	EGFR phosphorylation; ERK, AKT, CaMKII	ZR75-1, HCC1954, MDA-MB-415	Promote breast cancer cell proliferation and tumor growth, exhibiting correlation with disease grade and poor prognosis	(27)
	EGFR mRNA and protein expression	YMB-1, MDA-MB-453, MDA-MB-468, MDA-MB-231, MCF-7, Hs578T-Luc, BT-549	Contribute to tumorigenesis and metastasis	(208)
Gastric cancer	STAT6	MGC-803	Promote proliferation, migration, or invasion of GC cells	(209)
	SP1/MLL1	AGS, SGC7901	Contribute to invasion and migration of GC cells	(28)
Gastrointestinal cancer	PI3K/Akt	AGS, HGC27, HCT116, LOVO, HT29, MC38, CT26	Promote growth, metastasis, and invasion of GI cancer cells	(178)
Pancreatic cancer	EGFR phosphorylation; Ca2+	AsPC-1	Exhibit correlation with low patient survival probability	(177)
Head and neck cancer	EGFR protein expression	OE21, SCC4, BICR6, Te1, Te11, Te14, Te15	Form a functional complex with EGFR that jointly regulates HNSCC cell proliferation	(180)
	ERK1/2	UM-SCC1	Induce HNSCC cell proliferation and tumor growth	(143)
Hepatoma	ERK1/2,p38	SMMC-7721	Promote the cell proliferation, migration, invasion, and cell cycle progression	(185)
Prostate cancer	Regulation of p38 and JNK, phosphorylation	PC-3, DU145, LNCaP, 22RV1, VCaP	Promote cell growth, reduce apoptosis, and downregulate TNF-α expression in prostate cancer cells	(163)
Ovarian cancer	PI3K/Akt pathway	SKOV3, ES-2, Caov-3	Promote proliferation and invasion of ovarian cancer cells	(168)
Lung cancer	EGFR phosphorylation; MAPK signaling	H1299	Promote growth and invasion in lung cancer cells	(210)
		LA795	Participate in lung cancer cell proliferation, migration, and invasion	(187)
Non-small cell lung cancer	EGFR and STAT3	TH1975, PC9 and gefitinib-resistant PC9	Promote NSCLC cell proliferation and tumor growth	(211)
	EGFR and ERK1/2 phosphorylation	A549, H1993, HCC827, H1975, PC9	Promote the viability and migration of NSCLC cells	(212)

## Glossary

ACh: Acetylcholine
AD: Adenosine
ADCY1: Adenylate cyclase 1
AI: Artificial intelligence
AKT: Protein kinase B
AngII: Angiotensin II
ANO1: Anoctamin 1
ASM: Airway smooth muscle
ATP: Adenosine Triphosphate
CaCC: Calcium-activated chloride channel
CaCCs: Ca^2+^-activated Cl^−^ currents
CAMKII: Calmodulin-dependent protein kinase II
CaMKs: Ca^2+^/calmodulin (CaM)-dependent protein kinases
cAMP: Cyclic adenosine monophosphate
CCND1: Cyclin D1 gene
CF: Cystic fibrosis
CFTR: Cystic fibrosis transmembrane conductance regulator
CRC: Colorectal cancer
DRG: Dorsal root ganglia
EGFR: Epidermal growth factor receptor
EMS1: Cortactin gene
ER: Estrogen receptor
ERK: Extracellular signal-regulated kinase
ERK1/2: Extracellular-regulated kinases 1/2
ESCC: Esophageal squamous cell carcinoma
GI: Gastrointestinal
GIST: Gastrointestinal stromal tumors
GPCRs: G protein-coupled receptors
HER2: Human Epidermal Growth Factor Receptor 2
HNSCC: Head and neck squamous cell carcinoma
ICCs: Interstitial cells of Cajal
IP3: Inositol 1,4,5-triphosphate
IP3R /IP3R1: Inositol IP3 receptor /Inositol IP3 receptor 1
MAPK: Mitogen-activated protein kinase
MEK: Mitogen-activated protein
MONNA: N-(4-methoxy-2-naphthyl)-5-nitroanthranilic acid
MUC: Mucin
NF-κB: Nuclear factor kappa-B
NSP4: Non-structural protein 4
PASMCs: Pulmonary artery smooth muscle cells
PDAC: Pancreatic ductal adenocarcinoma
PH: Pulmonary hypertension
PI3K: Phosphatidylinositol 3-kinase
PIP_2_: Phospholipid phosphatidylinositol-4,5-bisphosphate
PLC: Phospholipase C
PR: Progesterone receptor
RAAS: Renin-angiotensin-aldosterone system
SHR: Spontaneous hypertensive rats
STT: Spontaneous tonic tension
TGF-β: Transforming growth factor β
TME: Tumor microenvironment
TRPV1: Transient receptor potential vanilloid 1
UICC: Urethral interstitial cells
USMCs: Urethral smooth muscle cells
VGCCs: Voltage-gated Ca^2+^ channels
VSMC: Vascular smooth muscle cell
5-HT: Serotonin
